# Prevalence and Characterization of Motile *Salmonella* in Commercial Layer Poultry Farms in Bangladesh

**DOI:** 10.1371/journal.pone.0035914

**Published:** 2012-04-25

**Authors:** Himel Barua, Paritosh K. Biswas, Katharina E. P. Olsen, Jens P. Christensen

**Affiliations:** 1 Department of Veterinary Disease Biology, Faculty of Life Sciences, University of Copenhagen, Frederiksberg, Copenhagen, Denmark; 2 Department of Microbiology, Faculty of Veterinary Medicine, Chittagong Veterinary and Animal Sciences University, Chittagong, Bangladesh; 3 The National Reference Laboratory for Enteropathogenic Bacteria, Department of Microbiological Diagnostics, Statens Serum Institut, Copenhagen S, Denmark; Indian Institute of Science, India

## Abstract

*Salmonella* is a globally widespread food-borne pathogen having major impact on public health. All motile serovars of *Salmonella enterica* of poultry origin are zoonotic, and contaminated meat and raw eggs are an important source to human infections. Information on the prevalence of *Salmonella* at farm/holding level, and the zoonotic serovars circulating in layer poultry in the South and South-East Asian countries including Bangladesh, where small-scale commercial farms are predominant, is limited. To investigate the prevalence of *Salmonella* at layer farm level, and to identify the prevalent serovars we conducted a cross-sectional survey by randomly selecting 500 commercial layer poultry farms in Bangladesh. Faecal samples from the selected farms were collected following standard procedure, and examined for the presence of *Salmonella* using conventional bacteriological procedures. Thirty isolates were randomly selected, from the ninety obtained from the survey, for serotyping and characterized further by plasmid profiling and pulsed-field gel electrophoresis (PFGE). Results of the survey showed that the prevalence of motile *Salmonella* at layer farm level was 18% (95% confidence interval 15–21%), and *Salmonella* Kentucky was identified to be the only serovar circulating in the study population. Plasmid analysis of the *S.* Kentucky and non-serotyped isolates revealed two distinct profiles with a variation of two different sizes (2.7 and 4.8 kb). PFGE of the 30 *S.* Kentucky and 30 non-serotyped isolates showed that all of them were clonally related because only one genotype and three subtypes were determined based on the variation in two or three bands. This is also the first report on the presence of any specific serovar of *Salmonella enterica* in poultry in Bangladesh.

## Introduction

*Salmonella* is a major food-borne pathogen worldwide and contaminated poultry products, especially undercooked meat and raw eggs are important sources of it [1], [2]. The rationality in introducing statutory surveillance for *Salmonella* in poultry farms/holdings in the EU member countries and other developed parts of the world is to reduce human salmonellosis of poultry origin [3]–[6]. In contrast, monitoring for *Salmonella* in poultry is either of very primitive type or the need is completely ignored in developing countries because of resource constraints, and therefore, information on its prevalence is poorly documented, so is the consequence to the public health. The zoonotic *Salmonella* circulating in developing countries with the possible presence of antimicrobial resistance genes might have some global public heath impacts because of their transmissions to other countries beyond the geographical origin, by travellers or by trades [7]–[10], are impossible to prevent. Mitigation of the source(s) at the geographical origin should be the option to restrain a wider dissemination of the zoonotic serovars for which local knowledge on their prevalence is important.

*Salmonella*, a member of Enterobacteriaceae consists of two species – *Salmonella enterica* and *Salmonella bongori*. *Salmonella enterica* consists of six subspecies (ssp.) under which there are >2500 serovars [11] that can produce diseases in mammals including animals and humans, and a good number of them can be harboured by poultry without showing any clinical signs [12]–[14]. *S. enterica* ssp. *enterica* serovar Gallinarum-Pullorum is host specific and non-motile and produce clinical diseases with variable mortality only in chickens [15]. Only motile serovars for which poultry are known to be reservoirs are zoonotic. Among them, most frequently reported serovars in the United States are *S.* Typhimurium, *S.* Enteritidis, *S.* Newport, *S.* Heidelberg and *S. enterica* ssp. *enterica* 4, [5], 12: i:- [16], although persistency and prevalence of different serovars vary from place to place [12].

Eggs produced from layer farms are a major protein source for people in Bangladesh and the spent hens are also sold for consumption. Small-scale commercial farms (FAO-defined production system 3) [17] are predominating here as in the other South and South-East Asian countries where stocks range from several hundreds to a few thousands, kept in a semi-confined system with a minimum of biosecurity. In such a system (FAO-defined production system 3), unlike large-scale commercial production systems (FAO-defined production systems 1 and 2) seen in developed countries, the birds might be more vulnerable to become exposed to *Salmonella*. However, published information on the rate at which small-scale layer farms are harbouring the zoonotic *Salmonella* in the South and South-East Asian countries including Bangladesh is limited, if not absent. Here, we describe the prevalence of zoonotic *Salmonella* at layer farm level in Bangladesh, the circulating serovars and the molecular characterization of these isolates.

## Materials and Methods

### Ethics statement

Oral permission was taken from owner of each poultry farm while collecting faecal samples from the farm.

### Study population

In Bangladesh, there are two districts (out of 64 districts) where the percentages of commercial layer poultry farms are the highest [18]. They are Dhaka and Chittagong where the capital city and the 2^nd^ largest city are located, respectively. By lottery we selected Chittagong, located in the South-East part, to conduct a cross-sectional survey for the prevalence of motile *Salmonella* at layer farm level. To provide state veterinary services to the public there are 64 districts and 481 sub-districts/Upazila (the lowest administrative unit in Bangladesh) livestock offices. We collected the list of all commercial layer poultry farms from the District Livestock Office. Using this list as the sampling frame, 500 farms were randomly selected. The sample size was estimated following the formula, n = Z^2^_1−α/2_ p (1−p)/L^2^, where n = number of sample size, p = prevalence of the disease, Z_1−α/2_ = value of the standard normal distribution corresponding to a two-sided confidence level of 1−α/2 and L = maximum allowable error. Because the farm prevalence of *Salmonella* in any commercial production system had not previously been documented in Bangladesh or in any South-East Asian country, expected flock prevalence was considered as 50% with an allowable error on the estimate of L = 0.05 at 95% confidence level.

### Collection of samples

The survey was conducted between July 2009 and June 2010. Each selected farm was physically visited once to collect pooled faecal samples and epidemiological information. Because most farms were single-housed we sampled one flock per farm. From five different locations in the farm, five naturally pooled faecal samples, each resulted from ∼30 cross-sectional pinches for achieving a total volume of about 200 g, were collected; no individual birds were sampled [19], [20]. Disposable plastic hand gloves were worn during sample collection. Each pooled sample was placed separately into a sterile plastic bag and transferred to the microbiology laboratory, Chittagong Veterinary and Animal Sciences University (ML-CVASU), Bangladesh at ambient temperature. After arrival at the laboratory the samples were stored at 5°C until examination.

### Isolation and identification of *Salmonella*

At ML-CVASU, for each sample a slurry was created by mixing 200 g of faeces with 200 ml of buffered peptone water (BPW; CM0009; Oxoid Ltd., England), and 50 g of this mixture was inoculated into 200 ml of BPW and incubated at 37°C for 18 hours. After that, 0.1 ml of this culture was inoculated into Rappaport-Vassiliadis (RV) broth (02-379; Scharlau Chemie S. A, EU), and incubated at 42°C for 48 hours; then 0.010 ml of the fresh culture was streaked on to brilliant green (BG) agar (CM0329; Oxoid Ltd., England) and Xylose-Lysine Deoxycholate (XLD) agar (CM0469; Oxoid Ltd., England) surface, and incubated overnight at 37°C. Suspected colonies from both of the agar plates were transferred to triple-sugar-iron (TSI) agar (CM277; Oxoid Ltd., England) slant and incubated at 37°C for 24 hours. Typical reactions for *Salmonella* to TSI were regarded as the presence of *Salmonella*. A farm was considered presumptively *Salmonella-*positive when ≥1 of the 5 collected samples were diagnosed positive with *Salmonella*. With accruing one isolate per positive farm over the period of the survey a repository was maintained at −80°C at ML-CVASU. At the end of the survey all the isolates were shipped to the Department of Veterinary Disease Biology, University of Copenhagen, Denmark (DVDB-KU) using Stuart's transport medium (CM111; Oxoid Ltd., England) at normal temperature by a commercial courier service. Upon receiving the samples at DVDB-KU, the isolates were screened for confirmation of motile *Salmonella*. Here, each isolate was grown on Luria Bertani (LB) broth (240230; Difco, USA) at 37°C and 100 µl of the overnight culture, divided into three separate and equally-spaced drops, was inoculated on to the surface of Modified Semisolid Rappaport Vassiliadis (MSRV) medium (CM0910; Oxoid Ltd., England) supplemented with novobiocin (SR0161E; Oxoid Ltd., England) and incubated at 41.5°C for 24 to 36 hours. Any swarming growth observed on the MSRV plates was transferred to brilliant-green phenol-red lactose sucrose (BPLS) agar (1.07237.0500; Merck, Germany) by dipping an inoculating loop into the swarmed zone. Following overnight incubation at 37°C, suspected *Salmonella* colonies from BPLS agar plates were transferred into 5% citrated blood agar (Blood agar base; CM0055; Oxoid Ltd., England) and incubated at 37°C for 16 to 18 hours. Standard biochemical tests were performed to assess the growth for *Salmonella* and isolates showing typical reactions were confirmed serologically using anti-*Salmonella* polyvalent serum (SSI, Copenhagen, Denmark), and stored at −80°C using 15% glycerol.

Thirty randomly selected isolates were serotyped according to White-Kauffmann-Le Minor Scheme [11] at Statens Serum Institiut, Copenhagen, Denmark. We used CE marked (ISO) *Salmonella* antisera (SSI Diagnostica, Hillerød, Denmark) for serotyping. PBS (pH 7.38) was used as a control to check for autoagglutination of the individual antiserum.

### Plasmid profiling

Plasmid was isolated according to the alkaline-lysis method described by Kado and Liu [21] with minor modifications [22]. Plasmids in *E. coli* 39R861 [23] and *E. coli* V517 [24] were used as references for standard plasmid sizes. The sizes of plasmids were estimated by calculating the migration of plasmid mobility relative to that of the reference plasmids [25].

### Genotyping of the isolates by pulsed-field gel electrophoresis (PFGE)

PFGE was performed following the standardized CDC PulseNet protocol [26] to determine the genetic diversity and relatedness among the isolates. Overnight culture of bacteria grown on brain heart infusion (BHI) broth (CM1135; Oxoid Ltd., England) was used. Genomic DNA was prepared using 1% agarose (SeaKem® gold agarose, Lonza, Rockland, ME USA) and embedded DNA was digested using 60U of the restriction enzyme *Xba*I (R0145L; New England BioLabs Inc.) for 14 hours at 37°C. The DNA fragments were isolated by electrophoresis in 0.5× TBE buffer using CHEF DR III (Bio-Rad Laboratories, Hercules, California, USA) system at 14°C with initial switch time 2.2 sec, final switch time 63.8 sec, current 6 V/cm, included angle 120 and run time 19 hours. *S.* Braenderup H9812 was used as a reference strain and as standard size marker [27]. The gel was stained with 1% ethidium bromide (E1510; Sigma-Aldrich, USA) solution for 30 minutes and destained in deionzed water for 3 times with 20 minutes interval. Using UV transillumination, gel image was captured by GelDoc EQ system with Quantity One® (Version 4.2.1) software (Bio-Rad Laboratories, Hercules, California, USA) and obtained images were saved in TIF format in computer. The analysis of the fingerprints was performed using GelCompar®II (version 4.6) software (Applied Maths, Belgium). Dice coefficient with a band position tolerance of 1% and 0.5% optimization level were used to determine similarity between fingerprints. The unweighted pair group method with arithmetic averages (UPGMA) was applied to produce the dendrogram. The DNA restriction patterns of the isolates were interpreted according to the criteria described by Tenovar *et al.* [28].

### Statistical analysis

All epidemiological data were entered into a spreadsheet of Microsoft Excel 2003 and transferred to statistical software SPSS (version 13.0, 2006) for windows (SPSS Inc., Chicago, IL). Farm-prevalence of *Salmonella* was calculated as the number of positive farms divided by total number of farms investigated. The difference in prevalence among different variables was shown using a χ^2^ test.

## Results

### Prevalence of *Salmonella* at layer farm level

An overall farm prevalence of motile *Salmonella* along with the prevalence seen with the variables: flock size, age, feed with animal protein, source of protein, use of antibiotics, and season are shown in Table 1. Of the 500 farms investigated 90 were positive for motile *Salmonella*, giving a prevalence of 18% (95% confidence interval (CI) 15–21%). The prevalence varied among the flock sizes; the farms of >1 but ≤2 thousand birds had the lowest prevalence compared with other groups (p<0.001). There was no significant difference observed on the prevalence of *Salmonella* in the farms having birds of different age groups (P = 0.48). None but one farm had a history of *Salmonella* vaccination. Commercially available feed or self-made feed, by mixing raw ingredients purchased from the local markets, were fed to the birds. The farms that had the history of using any kind of animal protein ingredient had a proportionately higher prevalence, although statistically borderline insignificant (P = 0.08) and the prevalence was ∼3 times higher (p<0.001) where fish meal was used compared with other protein sources. No significant difference was observed on the prevalence of *Salmonella* between farms using or not using antibiotics in feed as feed additives. The prevalence of *Salmonella* varied proportionately according to different seasons, although statistically not significant (P = 0.9).

**Table 1:** Prevalence of motile *Salmonella* in commercial layer poultry farms in Bangladesh, 2009–2010 (n = 500).

		No. farms investigated	No. positive with *Salmonella*	Prevalence (%)
Flock size*	≤1000	155	39	25.2
	1001–2000	195	17	8.7
	2001–4000	85	17	20.0
	>4000	65	17	26.2
Age of birds (wks)	≤20	135	21	15.6
	21–40	135	24	17.8
	41–60	105	17	16.2
	>60	125	28	22.4
Feed with animal protein	Yes	281	60	21.4
	No	58	7	12.1
	Unknown	161	23	14.3
Source of protein*	Fish meal	187	50	26.7
	Others	70	6	8.6
	Unknown	243	34	14.0
Use of antibiotics in feed	Yes	107	22	20.6
	No	279	56	20.1
	Unknown	114	12	10.5
Season	Summer (March–May)	114	23	20.2
	Rainy (June–August)	110	20	18.2
	Autumn (September–November)	125	22	17.6
	Winter (December–February)	151	25	16.6
Overall		500	90	18

* P<0.001.

### Serotyping and plasmid profiling

**Figure 1:**
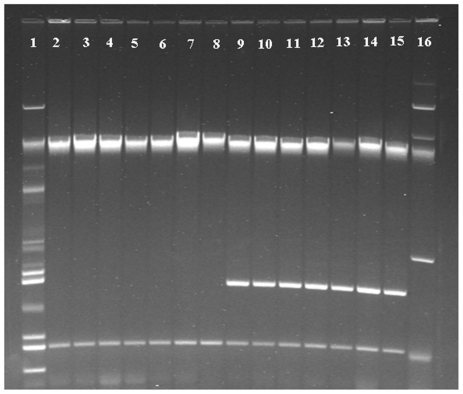
Plasmid profiles of 14 of the 30 *Salmonella* Kentucky isolates from commercial layer poultry farms in Bangladesh, 2009–2010. Lane 2–15 for *S.* Kentucky; Lane 1 and 16 are plasmid size markers in *Escherichia coli* strains V517 and 39R861, respectively (90 isolates: 30 *Salmonella* Kentucky and 60 non-serotyped were investigated).

The results of serotyping revealed that all of the 30 isolates belonged to the serovar *S.* Kentucky. We examined all the 90 isolates by plasmid profiling and the results of 14 *S.* Kentucky isolates are shown in Figure 1. Irrespective of serotypic identity two distinct profiles with two different sizes of plasmid were seen; 53 isolates harbored only one plasmid of 2.7 kb and 37 had two plasmids of 2.7 and 4.8 kb. Among the 30 *S.* Kentucky isolates 18 had one plasmid (2.7 kb) each and the others each had two plasmids (2.7 and 4.8 kb).

### PFGE genotyping

**Figure 2:**
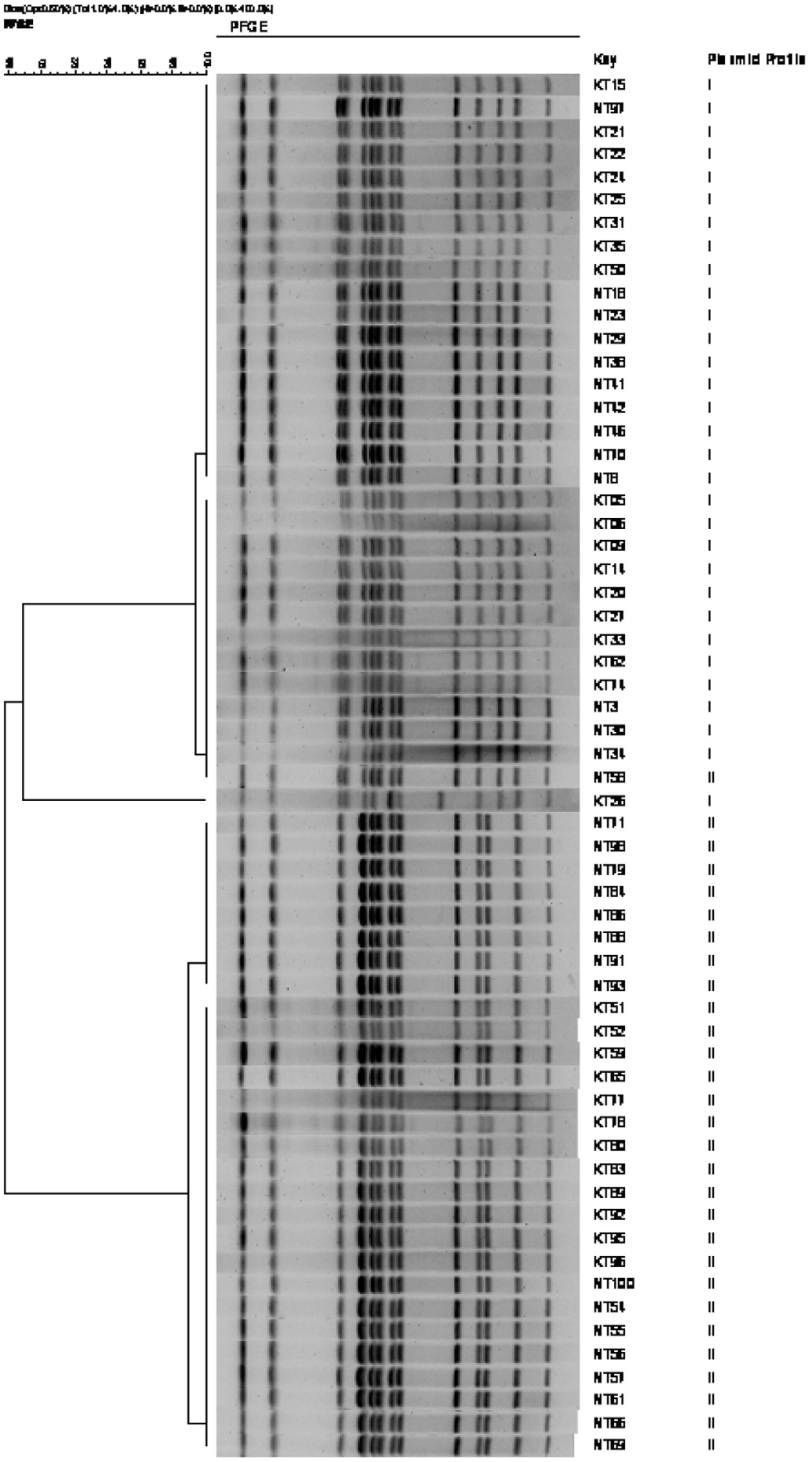
Dendrogram showing the cluster analysis on the basis of *Xba*I-PFGE of the 30 *Salmonella* Kentucky and 30 non-serotyped isolates obtained from commercial layer poultry farms in Bangladesh, 2009–2010. Dice coefficient was used to perform similarity analysis, and clustering was performed by using unweighted pair-group method with arithmetic means (UPGMA) with 1% band position tolerance and 0.5% optimization parameter. KT, *S.* Kentucky; NT, Non-serotyped.

PFGE typing demonstrated that all the *S.* Kentucky isolates were closely related, displaying a common band pattern. Three subtypes were identified based on the variations in two or three bands among the isolates tested. In addition to 30 *S.* Kentucky isolates, 30 randomly selected non-serotyped isolates were also subjected for genotyping and the results showed that their fingerprint patterns were similar to the *S.* Kentucky isolates. The dendrogram showing the restriction fingerprint pattern of the 60 isolates is illustrated in Figure 2.

## Discussion

In this survey the prevalence of motile *Salmonella* in commercial layer poultry farms in Bangladesh is 18%. Surprisingly, all of the 30 isolates investigated for serotyping belonged to the serovar *S.* Kentucky, and the fingerprint pattern of the other 30 non-serotyped isolates in PFGE analysis unraveled a similar identity, illustrating a high dissemination of *S.* Kentucky in layer farms in Bangladesh. However, because we investigated only one isolate per *Salmonella-*positive farm the circulation of other serovar(s) can not entirely be addressed. This is also the first report on the presence of any specific serovar in poultry in Bangladesh, and to the authors' knowledge, *S.* Kentucky has probably never been reported before in layer poultry from any other South and South-East Asian countries, although a report on prevalence of *Salmonella* belonging to serogroups B and D in poultry in a selected area in Bangladesh [29] is available.

We performed serotyping of the 30 isolates at Statens Serum Institut, which is a national reference laboratory in Denmark. The laboratory holds an accreditation according to DS/EN ISO/IEC 17025∶2000 for all the analyses. Therefore, we believe that the serotyping of the isolates reflects the true result. In addition, PFGE genotyping results echo the similar identity of the isolates.

Although RV broth was initially used, MSRV medium was used later as selective enrichment to ensure the growth of only motile *Salmonella*.

The dominance of one serovar over others in a particular geographical area is not uncommon [30], [31], although the presence of more than one zoonotic serovar of poultry origins have been reported frequently from well-structured surveillance carried out in the developed world [16], [32]. However, circulation of only one serotype in a spatial area is probably a rarity. Historically, *S.* Typhimurium and *S.* Enteritidis are two widely reported common zoonotic serovars associated with poultry, but none of them were found in this study. Other serovars have also been reported in different parts of the world – *S.* Paratyphi B var. Java in the Netherlands [33], *S.* Hiduddify in Nigeria [34], *S.* Infantis in Hungary [35], *S.* Hadar, *S.* Heidelberg, *S.* Manhattan and *S.* Virchow in Algeria [36], indicating temporal increase of a particular serovar in a specific region.

Although large flock size constitutes a potential risk factor for occurrence of *Salmonella* [37], [38], we observed an inconsistent result – farms with smallest flock size (<1000 birds) and the largest (>4000 birds) and the second largest (2001–4000 birds) had a prevalence of >20% while the farms of the second smallest flock size (1001–2000 birds) had the lowest prevalence, 9% (Table 1). It is hard to explain why such variations occur, however, because the prevalence was the same for the farms having flocks of the four different age groups (Table 1), a common exposure source for *Salmonella* to them might be a possibility, not just increasing excretion frequencies from carrier birds housed.

*S.* Kentucky was first reported by Edwards [39]. Poultry is considered a reservoir of this serovar, [7] which apparently is becoming more common [16] in chickens. It's presence in layer farms has been documented [31], [36], and the fraction of this serovar in broiler chickens has increased from 25% in 1997 to nearly 50% in 2006 in relation to top serovars identified in the USA [40]. Recently a particular clone of *S.* Kentucky acquiring a virulence plasmid from avian pathogenic *Escherichia coli* (APEC) has been described [41]. *S.* Kentucky was previously reported as a less successful pathogen in relation to human illness [8], however, evolving resistance in this serovar to multiple antibiotics, especially ciprofloxacin [7]–[9], [42], [43] are posing a new threat to public health.

Plasmid profiling is one of the earliest molecular tools used for subtyping *Salmonella* [44]. Two distinct profiles which shared a common small plasmid (2.7 kb) were seen in this study among the isolates, not consistent with Majtán *et al.* [9] who reported two large size plasmids in *S.* Kentucky isolates of human origin. Plasmid free strains of *S.* Kentucky have also been documented [43]. The plasmid contents of bacteria may change over time during storage [45]. For genotyping of *Salmonella*, PFGE is considered to be a gold standard test to investigate the epidemiology of outbreaks including source identification [46]. The band differences, seen in PFGE analysis, among the isolates investigated could be due to a single genetic event with a point mutation or an insertion or deletion [28], however, the overall results reveal that they all are clonally related. However, *S.* Kentucky has previously been shown to be highly clonal concerning population structure [47], and more discriminatory typing methods, e.g. multiple-locus variable-number tandem-repeats analysis (MLVA) may add new information to the epidemiology of the infection.

Animal protein sources used in poultry feed have been documented to be reservoirs of many serovars including *S.* Kentucky [48]–[50]. In this study, the prevalence was three times higher where fish meal was used. Experience on farm visits suggests that this raw ingredient is purchased from the local markets by the farmers themselves to produce low-cost feed by mixing with other ingredients. Animal protein added locally produced feeds are also used, but their *Salmonella*-free status is questionable because of absence of any structured surveillance and regulatory legislation.

Although prevalence of motile *Salmonella* was significantly associated with use of feed containing animal protein sources, especially fish meal, this practice was however not a commonality for the *S.* Kentucky positive farms. The farmers in the study area buy fish meal or other feed ingredients from the local markets where birds and eggs of different farms are also sold. The same vehicles are used for transportation of birds, eggs and feeds between the farms and the markets, and in most cases, these vehicles remain contaminated with faeces, and non-disinfected. The use of the same vehicles between farms and markets for transportation of birds, eggs and feeds, and the access of the products of the farms to the same local markets were two practice commonalities. Different degrees of faecal contaminations of vehicles and frequencies of market visits could have some contributory roles in the farm-positivity for *S.* Kentucky. This speculation however needs to be verified in future investigations.

The present study provides novel information on the prevalence of *Salmonella*, and circulation of a clonally related genotype of *S.* Kentucky in commercial layer poultry farms in Bangladesh. The high prevalence of this genotype should concern the authorities that it can be transmitted to humans by contaminated eggs. Tracing of the probable sources of the genotype is important to minimize its zoonotic risks within and outside of the country for which more molecular epidemiological studies are required to screen commercial feeds, raw feed ingredients, especially fish meal, and breeder farms, because *S.* Kentucky can also be vertically transmitted.

In conclusion, the prevalence of motile *Salmonella* in small-scale commercial layer poultry farms in Bangladesh is 18%, and only one serovar, *S.* Kentucky has been demonstrated so far. Based on plasmid profiling and PFGE analysis it is evident that the circulating isolates of the serovar is clonally related. This may suggest a common source of origin but more discriminatory typing methods may be able to add more information as to the epidemiology of *S.* Kentucky in Bangladesh. The high prevalence of the serovar might be attributable to some common sources of spread, not known from this study, but its emergence and persistency might have public health impacts in Bangladesh and beyond.
